# Compartmentalized Microhelices Prepared via Electrohydrodynamic Cojetting

**DOI:** 10.1002/advs.201800024

**Published:** 2018-04-19

**Authors:** Manjae Gil, Seongjun Moon, Jaewon Yoon, Sahar Rhamani, Jae‐Won Shin, Kyung Jin Lee, Joerg Lahann

**Affiliations:** ^1^ Department of Fine Chemical Engineering and Applied Chemistry College of Engineering Chungnam National University 99 Daehak‐ro (st) Yuseong‐gu Daejeon 305‐764 Republic of Korea; ^2^ Macromolecular Science and Engineering University of Michigan Ann Arbor MI 48109 USA; ^3^ Department of Biomedical Engineering University of Michigan Ann Arbor MI 48109 USA; ^4^ Institute of Functional Interfaces Karlsruhe Institute of Technology 76344 Eggenstein‐Leopoldshafen Germany; ^5^ Department of Chemical Engineering University of Michigan Ann Arbor MI 48109 USA

**Keywords:** anisotropic particles, biomimetic materials, electrohydrodynamic cojetting, helical microstructures, patchy surfaces

## Abstract

Anisotropically compartmentalized microparticles have attracted increasing interest in areas ranging from sensing, drug delivery, and catalysis to microactuators. Herein, a facile method is reported for the preparation of helically decorated microbuilding blocks, using a modified electrohydrodynamic cojetting method. Bicompartmental microfibers are twisted in situ, during electrojetting, resulting in helical microfibers. Subsequent cryosectioning of aligned fiber bundles provides access to helically decorated microcylinders. The unique helical structure endows the microfibers/microcylinders with several novel functions such as translational motion in response to rotating magnetic fields. Finally, microspheres with helically patterned compartments are obtained after interfacially driven shape shifting of helically decorated microcylinders.

## 1 Introduction

Anisotropically compartmentalized microbuilding blocks have attained significant attention recently due to their unique interfacial features and their potential applications, which are impossible to address with isotropic microparticles.^1^, ^2^ Specific applications range from sensors,^3^ drug delivery vehicles,^4^ surfactants,^2^, ^5^ and catalysts^6^ to soft microactuators.^7^ This progress has been enabled by an arsenal of methods for the fabrication of compartmentalized microparticles have been developed, such as stop‐flow lithography by Doyle and co‐workers,^8^ droplet microfluidics by Weitz's groups,^9^ or the PRINT method by DeSimone and co‐workers.^10^ Similarly, electrohydrodynamic (EHD) cojetting has been widely used for preparing multicompartmental microbuilding blocks.^2^, ^3^, ^4^, ^5^, ^6^, ^7^, ^11^, ^12^, ^13^

One of the more attractive advantages of multicompartmental microparticles is that they provide an opportunity in which to mimic biological systems and replicate complex biological functions in abiotic materials. Most of the micro‐organisms create unique patterns in their bodies, such as helical architectures.^14^ In terms of engineering, biological systems have been inspiring because of their potential for useful applications.^14^ For example, a paramecium can swim in water very effectively using helical patterned cilia. In addition, helical morphologies are of fundamental scientific interest because a better understanding of physical and chemical behaviors of helical structures can provide crucial clues to understanding biological systems. Nevertheless, the realization of helically patterned, synthetic multicompartmental microbuilding blocks have been limited to a small number of techniques, such as E‐beam lithography^15^ or block copolymer self‐assembly methods.^16^

Herein, we demonstrate a facile and scalable method for the preparation of helically decorated microbuilding blocks, using a modified EHD cojetting approach. In this one‐step approach, Janus microfibers are twisted during electrojetting resulting in bundles of helical microfibers. As needed, the collected microfiber bundles can then be converted into helically patterned microcylinders using cryosectioning. Importantly, diverse materials such as magnetic nanoparticles can be helically patterned in these microcylinders. In addition, compartmentalized microcylinders can be spatially decorated with molecules of interest. In a further extension of this approach, microspheres with uniquely patterned surfaces can be obtained from helically patterned microcylinders via interfacially driven shape shifting.^7^

## 2 Results and Discussion

The experimental setup used for EHD cojetting has been previously reported for the fabrication of Janus (bicompartmental) microfibers.^12^ In order to ensure the continuity of process, different polymeric solutions comprised of poly(lactic‐*co*‐glycolic acid) (PLGA), 85% glycolic acid (*M*_w_ 50 000–75 000 g mol^−1^), dissolved in a mixture of chloroform and dimethylformamide (DMF) with certain viscosity are processed through parallel needles arranged in a side‐by‐side configuration. The electrical field was adjusted until a straight polymer jet maintaining laminar flow was maintained (typically 10–15 kV). During electrojetting, the polymer jet was twisted by a rotating counter electrode connected to an external motor.

**Figure 1:**
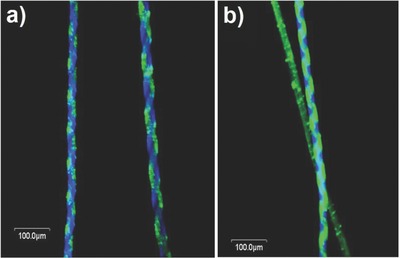
CLSM images of helically compartmentalized microfibers with a) left and b) right handedness (diameter: 30 µm, pitch size: 100 µm).

**Figure** **1**a,b presents confocal laser scanning microscopy (CLSM) images of Janus microfibers with helical intrastructures (twisting fiber jet‐stream from a side‐by‐side needle). To produce this microfiber, 40–50 wt% of PLGA dissolved in mixture of 1:9 v/v DMF and chloroform was used. The green and blue emission, which comes from organic dyes dissolved into each polymeric solution for visualization purposes, are maintained helically without an inner mixture, meaning the EHD cojetting and twisting procedures were successful. Obviously, because we use ex situ twisting, left and right handiness in helicity can be readily controlled, as shown in Figure 1 and Figure S1 in the Supporting Information (3D CLSM image of helical fibers). In addition, the pitch size of the helix can also be easily controlled by adjusting the rotations per minute (RPM) of twisting motors and twisting time. In Figure S2 in the Supporting Information, depicts merged fluorescence images of helical microfibers with different pitch sizes.

**Figure 2:**
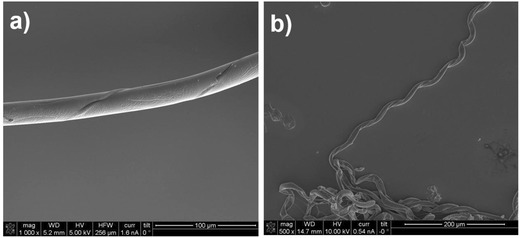
SEM images of a) microfibers with helical rails on wall (preparation of twisted core/shell fibers, followed by core etching) and b) microsprings (preparation of twisted core/shell fibers, and crosslinking of core compartment, followed by shell etching) (diameter: 30 µm, pitch size: 100 µm).

In principle, the underlying concept is rather generic and can lend itself to different jet morphologies including core/shell or even dual‐core/shell configurations.^7^, ^13^, ^17^ Earlier work by our group established that the overall jetting behavior of core–shell jetting is determined by the shell flow resulting in excellent jet stability comparable to jets obtained in the side‐by‐side configuration.^7^, ^12^ **Figure** **2**a shows microfibers that have helical rails on the fiber wall. Here, core–shell jetting has been performed by introducing the PLGA solution into the shell and the polyethyleneoxide (PEO) solution into the core, followed by subsequent motorized twisting. After twisting, the PEO compartments are selectively etched out by deionized (DI) water, resulting in the formation of helical rails onto the fibers because PEO can be easily dissolved in water. Although symmetric core/shell needles were used in these experiments, the core compartments generally tend to be positioned out of the center because of the complex hydrodynamics during jetting, which results in a helical rail on the wall, and not, as expected, a helical fiber core.

In addition, the opposite process is also possible, as described in Figure 2b. Here, a PVCi solution, which is a photo‐crosslinkable polymer, was introduced into the core needle (with the same shell system, i.e., PLGA). The core–shell fiber is first twisted and the core compartment is subsequently crosslinked by UV‐irradiation, having resistance to dissolution. Finally, the shell compartment is removed by solvent treatment, resulting in a microspring composed of the neat core compartment (here, PVCi). Both pitch size and handiness of the helical structures and microsprings are controllable by adjusting the twisting procedure. The thickness of those can be changed as well by controlling jetting parameters such as feed ratio and concentration of polymeric solution as described in our previous reports.^7^, ^17^

**Figure 3:**
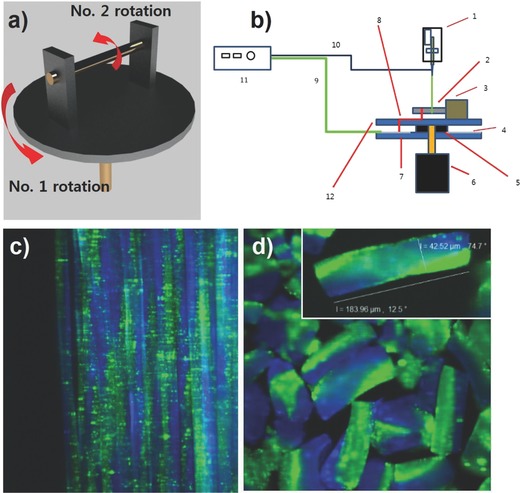
a) Schematic diagram of apparatus for preparing helical microfiber bundles in situ; RPM of No. 1 and No. 2 rotors was 1000–6000 and 10–100 RPM, respectively. b) Blueprint of apparatus: 1) syringe pump, 2) collector, 3) motor for No. 2 axis, 4) aluminum foil for electrical conduction of ground electrode, 5. bearing, 6. motor for No. 1 axis, 7) substrate for insulation, 8,9) wire for ground, 10) wire for power supply, 11) power supply, and 12) substrate for rotation. c) CLSM image of helical microfiber bundles. d) CLSM image of helical microcylinders obtained by sectioning microfiber bundles (sectioned by ≈200 µm) (diameter: 43 µm, pitch size: 200 µm).

When microfiber bundles with reasonable alignment are obtained, microcylinders can be produced by cryosectioning of the fiber bundles. Because the inner architecture of the microcylinders prepared by such a process will be identical to that of the original microfibers, microcylinders with helical microstructures can be prepared, if an in situ process for collecting helical fiber bundles is available. **Figure** **3**a,b shows the schematic illustration and blueprint of an in situ collecting apparatus to create helical microfiber bundles. This collector contains two rotating axes: one (No. 2 rotator in Figure 3a) for collecting fibers as a bundle shape and the other (No. 1 rotator in Figure 3a) for twisting fibers during jetting. The RPM of the first rotator should be about 100 times faster than that of the second rotator, considering our typical collecting speed for microfiber bundle and pitch size. This also means that the RPM of the first rotator can be used to control the desired pitch size of the helical microfibers. In order to demonstrate the effect of the RPM, we controlled the RPM of rotor 1 from 1000 to 6000 rpm. As shown in Figures S3 and S4 in the Supporting Information, when the helicity of twisting nanofiber is increased, the pitch distance of the fiber is also decreased, and the diameter of nanofiber was decreased as increasing the RPM of rotor 1. Thus, the adjust of RPM of rotor 1 can control of the structure of twisting nanofiber maintaining the continuity of nanofiber in the EHD process. The handedness of helical microfibers can be also controlled by adjusting the rotating direction of the first rotator. Figure 3c shows CLSM images of helical microfiber bundles collected by the in situ twisting setup (see also, Movie S1, Supporting Information). Using this approach, microfibers with a helical internal architecture can be obtained, and the compartment and shape of each microfiber is completely maintained during the twisting and EHD cojetting procedure, as shown in Movie S1 in the Supporting Information. This means that these jetting behaviors can be used when as external force is applied, implying that these can potentially be used in 3D printing applications.^18^ As described earlier, once microfiber bundles were obtained, microcylinders with desired lengths can be produced (Figure 3d). The microcylinders with 100 µm length contains several pitches in Figure 3d, but theoretically, we can obtain microcylinders having helical internal architecture with a desired length and pitch size, and with desired compartments as well by adjusting various experimental conditions, such as the number of starting laminar flows.^12^ It is quite meaningful that multicompartmental microcylinders with distinct internal architecture can be fabricated by EHD cojetting, which makes it possible to extend the EHD cojetting method to diverse application fields. The followings are several representative examples of these potential applications.

**Figure 4:**
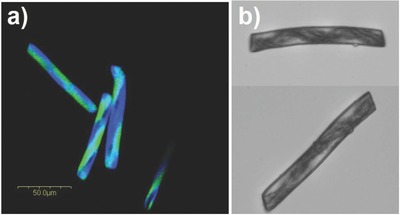
a) CLSM and b) optical microscopy images of microcylinders having inner helical architecture with magnetic nanoparticles in one compartment. The cylinders are sectioned at 70 µm. Optical microscopy images show that the pitch size of the helical structure can be controlled.

Because EHD cojetting involves the processing of polymeric solutions, diverse types of inorganic substances (additives) can be introduced into one of the compartments, if these materials are soluble (or dispersible) in organic solutions. The extension of the above‐described process to nanoparticle suspensions allows for preparation of microcylinders decorated with helical patterns of magnetic nanoparticles (Movie S2, Supporting Information). **Figure** **4** shows CLSM and optical microscopy images of helical microcylinders containing magnetic nanoparticles in one compartment with a helical manner. This work indicates a high level of can control over the handiness of the inner helical structure as well as the helical pitch size. In addition, these microcylinders can undergo controlled movement under the influence of an external magnetic field (Movie S2, Supporting Information). These magnetic microcylinders may provide novel insights in the relationship between their actual response against external stimuli, in terms of their internal architecture, and the corresponding shape/alignment of the magnetic field with potential implications for diverse applications such as the movement of helical swimmers.^19^

**Figure 5:**
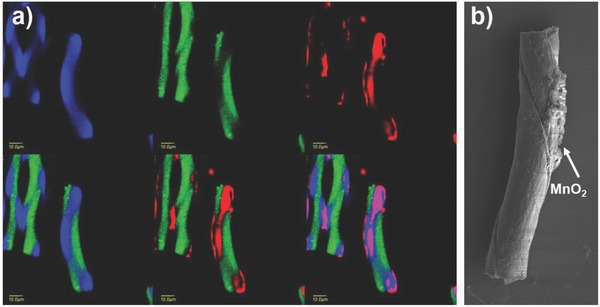
a) CLSM images of helical microcylinders decorated with a red dye in one compartment. b) SEM image of helical microcylinders decorated with MnO_2_ nanoparticles on one compartment (the length of microcylinder is identical with that of microcylinder provided in Figure 5a).

Second, because these helical microcylinders can be designed using different types of polymers in each compartment, different surface functionalities can be introduced onto cylinders in a helical manner. **Figure** **5**a shows CLSM images of helical microcylinders decorated with certain organic dyes onto one compartment. The original helical microcylinders are made of a compartment containing a carboxylated PLGA (visualized by a blue dye) and a compartment containing normal PLGA (green dye). To selectively modify the surface of these helical cylinders, amine‐polyethylene glycol (PEG)‐Rhodamine (red dye) was immobilized to the blue compartment through ethyl(dimethylaminopropyl) carbodiimide (EDC)/*N*‐hydroxysulfosuccinimide (sulfo‐NHS) coupling with the carboxyl groups in the PLGA polymer. Figure 5a demonstrates the CLSM analysis of these cylinders, where the overlay images clearly show the red dye on the blue compartment. This method can be extended to decorate microcylinders in a helical manner with such materials as metal nanoparticles. Figure 5b presents scanning electron microscopy (SEM) images of helical microcylinders, having helical decorated MnO_2_ nanoparticles on one compartment. Initially, MnCl_2_ is introduced into the buffer solution containing microcylinders, so that the Mn ion can be selectively anchored with COOH groups by ionic interaction, followed by chemical reduction. These methods essentially offer diverse possibility in their applications (i.e., production of microcylinders having a polymeric brush with a helical manner to mimic a *paramecium* or Christmas tree worm). Theoretically, we can consider a broad range of reactive, functional PLGA polymers during jetting, as described earlier, and thus diverse types of chemical reactions for spatioselective (or orthogonal) surface functionalization can be considered.^20^

**Figure 6:**
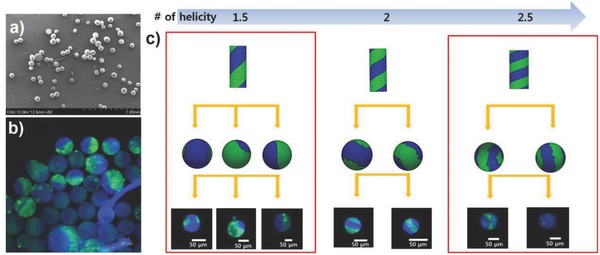
a) SEM image of microspheres prepared by shape evolution of helical microcylinders. b) CLSM image of helical microspheres. c) Schematic illustration of helical microcylinders with various pitch sizes (top row) and corresponding microspheres prepared by shape evolution (middle row, viewed from different angle), which is calculated by the Surface Evolver program. CLSM images of experimental data are also demonstrated according to their matched inner architecture (bottom row).

Finally, multicompartmental microcylinders with a helical inner architecture can evolve into microspheres with a distinct inner compartmentalization. It is difficult to imagine that this type of microspheres at this specific size and scale would be accessible through another synthetic method. Previously, we have demonstrated the successful shape‐shifting of microcylinders in response to external stimuli to produce multicompartmental microspheres.^7^, ^13^ When the polymeric microcylinders are heated over their *T*_g_ temperature in solution, the cylinders tend to change into a spherical shape, while maintaining their internal architecture due to surface tension.^7^ **Figure** **6**a,b presents SEM and CLSM images of microspheres after the shape evolution of helical microcylinders. The size and distribution of particles are dependent upon the initial microcylinders. Because the microcylinders are prepared by a top‐down approach with a well‐defined length and diameter, the final microspheres are relatively uniform in shape and size, and the inner architecture is maintained (Figure 6b). The internal architecture of microspheres will be dependent upon that of the initial microcylinders, and theoretically, we can control the pitch size and handedness of the helix as well as the inner architecture of the microspheres. Here, we compare the theoretical results of the inner architecture with respect to that of the initial microcylinders using the Surface Evolver simulation program, which provides a powerful tool to calculate surface energy and the corresponding results in shapes. (Detailed calculation procedure is described in the Supporting Information.)^21^ Figure 6c shows a schematic sketch for the theoretical results and a CLSM image of the experimental results. Microspheres with diverse inner architectures can be fabricated by adjusting the pitch size of the starting helical microcylinders. Combining the different methods, particle designers can produce multifunctional microparticles which contain a unique inner architecture with desired materials and tailored surface functionalities as well.

## 3 Conclusion

In summary, polymeric microbuilding blocks with helical surface patterns and inner architectures were successfully prepared via EHD cojetting using an in situ twisting method. Defining features of the helical microstructures can be easily controlled by adjusting the twisting procedure. In addition, different types of additives can be introduced into helical microstructures or different surface decorations on the helical microstructures can also be achieved. Helical microcylinders have also been obtained by cryosectioning of microfiber bundles and these microcylinders can be shape shifted by external stimuli, resulting in microspheres with distinct compartmentalization and unique microstructures. These techniques can provide versatile methods for the preparation of helical microstructures and can be extended to mimic biological microcreatures.

## 4 Experimental Section

*Materials*: Poly(d,l‐lactide‐*co*‐glycolide) (ester terminated, *M*_w_ 50 000–75 000 g mol^−1^) (PLGA) (Product No. is 430471‐5G), Poly [(*m*‐phenylenevinylene)‐*alt*‐(2,5‐dihexyloxy‐*p*‐phenylenevinylene)] (MEHPV), poly [tris(2,5‐bis(hexyloxy)‐1,4‐phenylenevinylene)‐*alt*‐(1,3‐phenylenevinylene) (PTDPV), phosphate buffered saline (PBS), and *N*‐(3‐dimethylaminopropyl)‐*N*'‐EDC were purchased from Sigma‐Aldrich, USA. Sulfo‐NHS was purchased from Thermo‐Fisher Scientific, USA. Amine‐PEG‐Rhodamine with a molecular weight of 3400 Da was purchased from Nanocs.

*Preparation of Helical Microfibers and Microcylinders by EHD Cojetting and Sectioning Procedure, and Shape Evolution of Microcylinders into Microspheres*: Typically, the EHD cojetting process with a side‐by‐side needle is identical to our precedent works,^12^ except for the collecting system. Previously, the rotating motor with an RPM of 30–60 is adopted in order to collect the multicompartmental fiber jet in a well‐aligned manner. Here, the additional rotating axis for the twisting jet stream of microfibers was introduced as described above. The second rotor acts as continuously collecting microfiber, and the first rotor is used to produce the helicity of microfiber. Thus, a continuous twisted microfiber can be prepared. For instance, the microfibers in Figure 1 were fabricated using 40–50 wt% of PLGA in 1:9 volumetric mixtures of DMF and Chloroform, 1000 RPM of rotor 1 and 60 RPM of rotor 2. The obtained microfibers have 30 µm of dimeters and 100 µm of pitch size. Fiber bundles were successfully collected without any deterioration of the inner architecture. The fiber bundles were embedded into the optimum cutting temperature (OCT) gel solution for cryosectioning and microcylinders with a desired length can be obtained. Separated microcylinders can be collected after mild sonication and shape evolution (or shape shifting) of these microcylinders into microspheres by applying external stimuli (either heat or sonication) has been carried out.

*Selective Surface Modification of Helical Cylinders*: After fabrication and sectioning, the helical cylinders, with carboxyl‐PLGA in one compartment, were washed several times with DI water to remove all impurities before being suspended in a PBS buffer with 1% v/v Tween 20. To activate the carboxyl groups for the attachment with amine‐PEG‐Rhodamine, the particles were incubated with 1 mmol EDC for 10 min, followed by 0.1 mmol sulfo‐NHS for 10 min. Upon activation of the carboxyl groups, the cylinders were incubated with 0.1 mmol of amine‐PEG‐Rhodamine and rotated for 2 h to complete the reaction and immobilize the dye selectively to the surface. The cylinders were then washed numerous times to remove all unreacted material before imaging with a CLSM to determine the selectivity of the immobilization.

*Characterization*: In case of CLSM, the images were collected using LSM5 LIVE from ZEISS. To obtain separated color, MEHPV, PTDPV, and amine‐PEG‐Rhodamine were used to represent blue color, green color, and red color, respectively. In addition, the SEM images were collected using S‐4800 Field Emission SEM from HITACH corp.

## Conflict of Interest

The authors declare no conflict of interest.

## Supporting information

Supplementary

Supplementary

Supplementary
