# Supplementary material for: Compartmentalized Microhelices Prepared via Electrohydrodynamic Cojetting
Source: Adv Sci (Weinh). 2018 Apr 19;5(6):1800024. doi: 10.1002/advs.201800024 (PMC6009775; doi:10.1002/advs.201800024)
Supplement: Supplementary file 1 — Supplementary [file ADVS-5-1800024-s002.pdf]

Copyright WILEY-VCH Verlag GmbH & Co. KGaA, 69469 Weinheim, Germany, 2018.

ADVANCED SCIENCE
Open Access

Supporting Information

for *Adv. Sci.,* DOI: 10.1002/advs.201800024

Compartmentalized Microhelices Prepared via Electrohydrodynamic Cojetting

Manjae Gil, Seongjun Moon, Jaewon Yoon, Sahar Rhamani, Jae-Won Shin, Kyung Jin Lee, and Joerg Lahann**

Copyright WILEY-VCH Verlag GmbH & Co. KGaA, 69469 Weinheim, Germany, 2018.

Supporting Information

Compartmentalized Microhelices prepared via Electrohydrodynamic co-Jetting

Manjae Gil, Seongjun Moon, Jaewon Yoon, Sahar Rhamani, Jae-Won Shin, Kyung Jin Lee and Joerg Lahann**

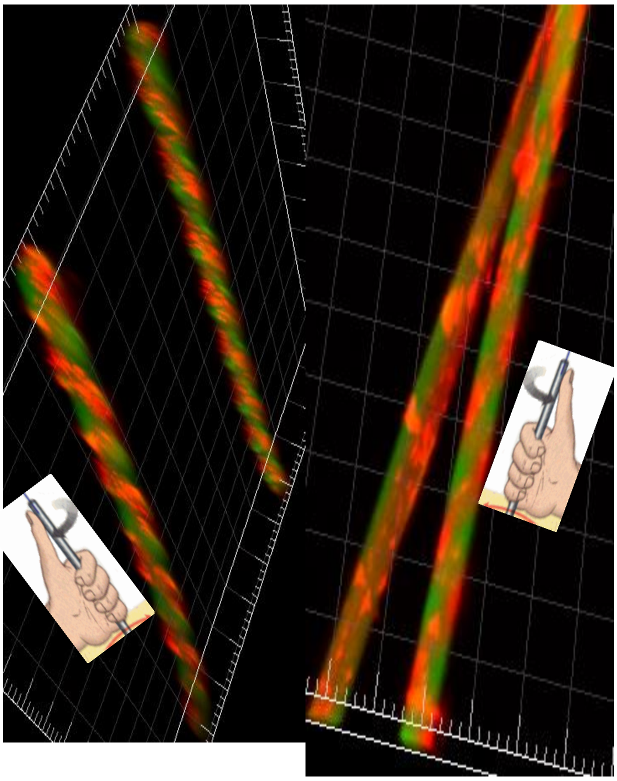

Figure S1. 3D CLSM image of helical nanofiber with right and left helix

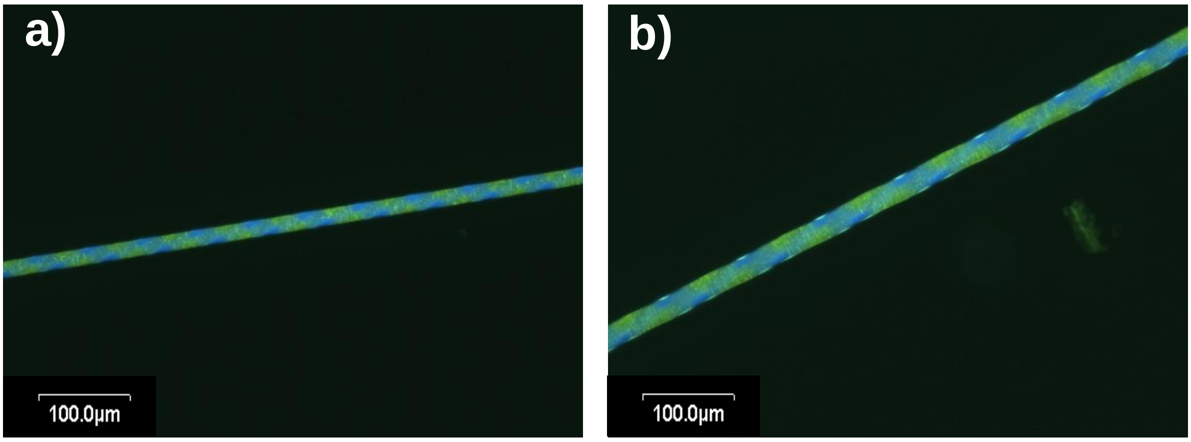

Figure S2. Fluorescence microscope image of microfiber with helican inner-architecture of different pitch size: about a) 50 µm and b) 100 µm

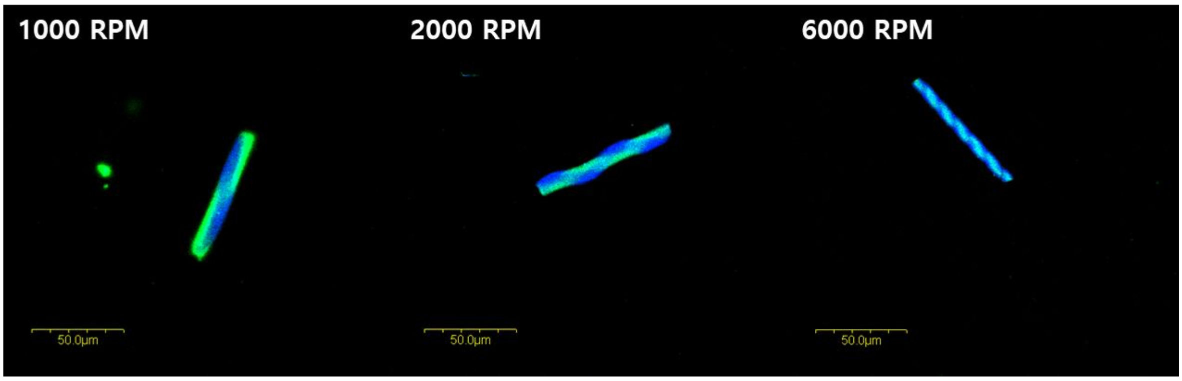

Figure S3. Fluorescence microscope image of micro-cylinder with different pitch size and helicity depending on the RPM of rotor 1

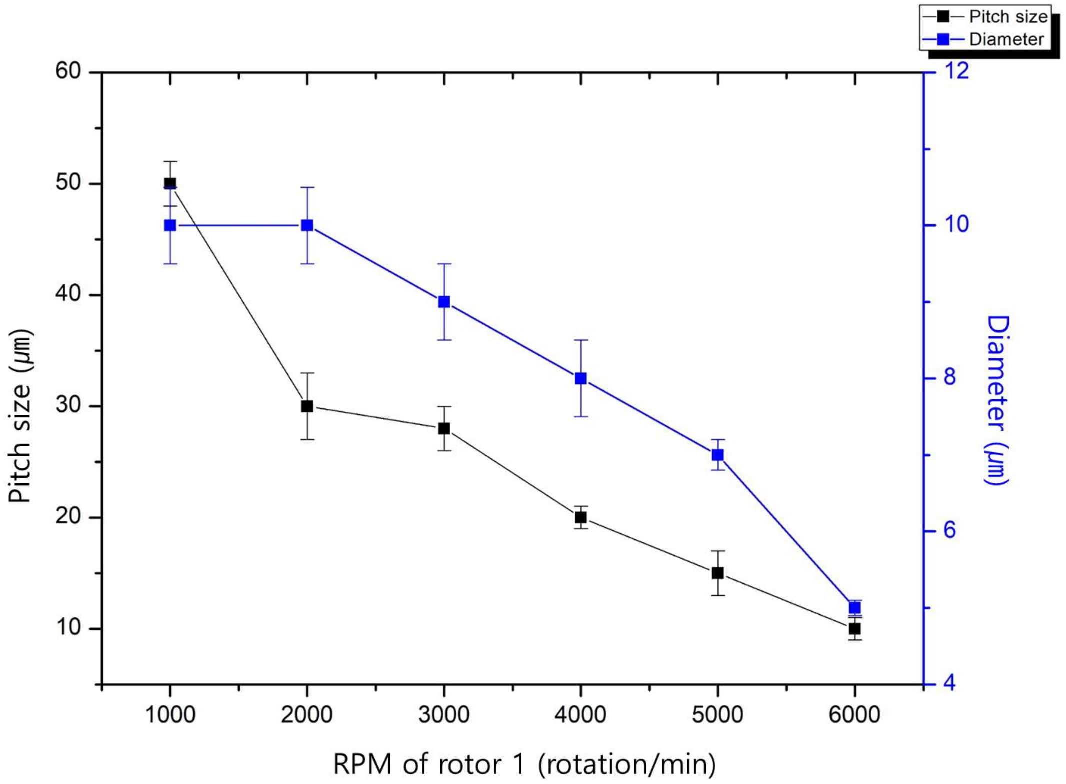

Figure S4. Quantitative relationship curves of diameter and pitch size depending on the RPM of rotor 1

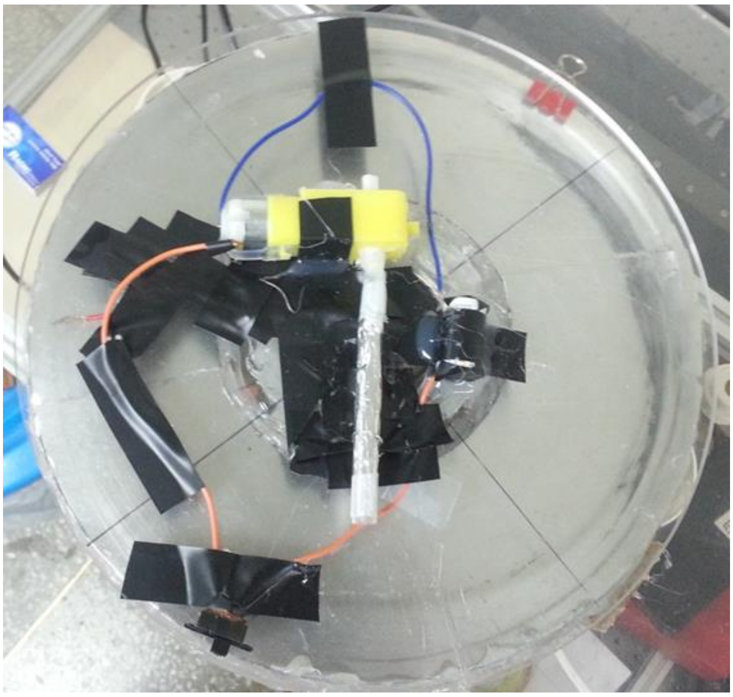

Figure S5. Photograph of in-situ twisting apparotus

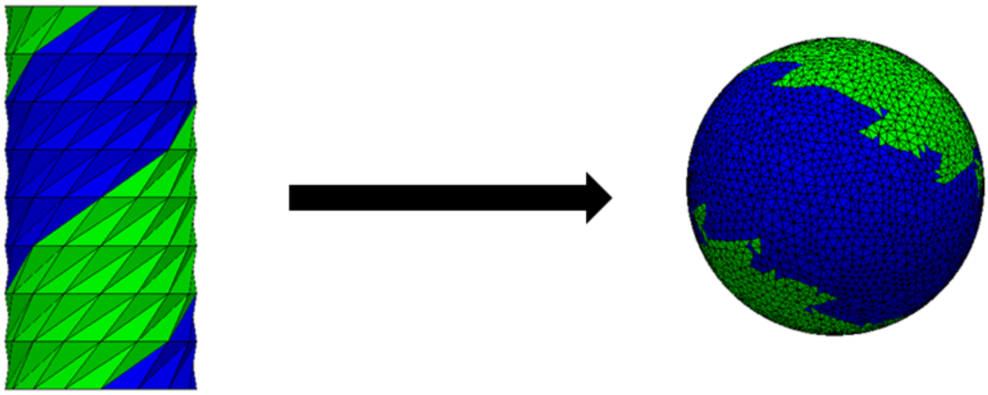

Figure S6. Schematic illustration of helical microcylinder with 1.5 helicity, and corresponding microspheres after calculating from surface evolver™

Movie S1. Movie for in-situ twisting process during EHD co-jetting

Movie S2. Movement of microcylinders containing magnetic nanoparticles with helical manner against external magnetic fields

Estimate the particle shape using surface evolver: We could find that the micro-cylinder, fabricated using PLGA, was melted, and they changed their structure from cylinder to spherical form to minimize its surface energy when the mirco-cylinders were located in high temperature water medium. Here, we used the simulating program to explain the morphological changes depending on helicity of the microcylinder. Firstly, we drew the helical cylindrical object as described in manual of surface evolver. To express the helical cylinder, we specified the vertexes, edges, and faces of helical cylinder respectively. And then, we could obtain the helical cylinder shape obtained from the experiment by applying helicity to the surface. Finally, through the simulating program, we can obtain the spherical structure after minimizing the surface energy depending on the helicity of microcylinder. As a representative example, we attached the file S1 which is the data file including the specific values for microcylinder with 1.5 helicity in Figure 6C. The detailed object of microcylinder can be confirmed when the file is opened using SE-fit (Figure S6) - the extension program of the surface evolver™. When mash making is done using surface evolver, it changes to spheres to minimize the surface energy of object, and we can predict the desired structure as shown right panel of Figure S6.

File S1. Surface evolver example data file (1.5 helicity)
